# Data from crosslinking and analysis of cDNAs (CRAC) of Nab3 in yeast cells expressing a circular ncRNA decoy

**DOI:** 10.1016/j.dib.2021.106951

**Published:** 2021-03-15

**Authors:** Tommaso Villa, Yan Jaszczyszyn, Domenico Libri

**Affiliations:** a Université de Paris, CNRS, Institut Jacques Monod, F-75006, Paris, France; b Institute for Integrative Biology of the Cell (I2BC), CEA, CNRS, Paris-Saclay University, Gif-sur-Yvette, France

**Keywords:** Pervasive transcription, Non-coding RNAs, Circular RNA, Transcription termination, Nrd1-Nab3-Sen1 (NNS) complex, Cryptic unstable transcripts, Yeast *Saccharomyces cerevisiae*, CRAC

## Abstract

Pervasive transcription originating from the ubiquitous activity of RNA Polymerase II (RNAPII) generates a vast mass of non-coding RNAs (ncRNAs) that represent a potential harm to gene expression. In the compact genome of the yeast *Saccharomyces cerevisiae*, the main genomewide safeguard against pervasive ncRNAs is the Nrd1-Nab3-Sen1 (NNS) complex, composed of two RNA-binding proteins (Nrd1 and Nab3) and the helicase Sen1. The NNS complex directs transcription termination of ncRNA genes and promotes the rapid degradation of pervasive transcripts from yeast nuclei through its physical and functional coupling to the nuclear RNA exosome. We have recently shown that inhibition of the exosome in yeast cells leads to the accumulation of ncRNAs complexed with Nab3 and Nrd1, decreasing recycling of these termination factors to sites of transcription and inducing global termination defects at NNS targets. Consistent with the notion that ncRNAs out-titrate Nab3 and Nrd1 termination factors, we have shown that a similar genomewide termination impairment could be achieved by expressing a circular RNA decoy containing a Nab3 binding target [1]. In relation to this previous research article, here we expand our observations on the effect of the circular RNA decoy on NNS termination. We aimed at verifying that the Nab3 binding sequence present on the decoy is indeed efficiently sequestering Nab3 as intended by design, leading to the expected decrease of Nab3 binding on NNS targets. We employed the crosslinking and cDNA analysis protocol (CRAC) on yeast cells expressing the circular ncRNA decoy or a control construct. We present data from high-resolution genomewide RNA binding of Nab3 in three independent biological replicates of these *S.cerevisiae* cells, normalized by spiked-in *S.pombe* lysates. These data allow the useful assessment of the extent of co-transcriptional binding decrease of Nab3 by decoy ncRNA titration and will be valuable for further analyses of NNS targeting mechanisms.

## Specifications Table

**Table:** 

Subject	Biology
Specific subject area	Molecular Biology, Genetics, Genomewide RNA-binding protein mapping (CRAC)
Type of data	Image Chart Figure
How data were acquired	Single-end read of cDNAs generated from Nab3-crosslinked RNA selected after stringent immunopurification. High-throughput sequencing performed with Illumina NextSeq 500 System.
Data format	Raw Analyzed Filtered
Parameters for data collection	Yeast strains expressing the decoy or control constructs under control of the Tet_OFF_ promoter were grown in CSM-URA-TRP medium supplemented with 2 μg/ml Doxycycline at 30 °C to mid-log phase, back diluted to early-log phase in the same medium without Doxycycline and grown to OD_600_ of 0.6 before being subjected to UV crosslinking in vivo and subsequently processed for CRAC.
Description of data collection	Three independent biological replicates per sample were UV-crosslinked in vivo, cryolyzed, spiked-in with *S.pombe* lysates, and subjected to a stringent native pull-down followed by a subsequent denaturing immunopurification with in-column adapters ligation. Proteins were isolated on a Gelfree 8100 system, RNA recovered, reverse-transcribed, and PCR amplified. The Nab3 binding dataset was collected from multiplexed single-end sequencing of cDNA libraries using Illumina NextSeq 500 System with an average of 50 million single 92 bp reads obtained per sample after demultiplexing. The raw reads were recorded in fastQ files, quality filtered, trimmed and mapped to reference yeast genome (R64).
Data source location	Université de Paris, CNRS, Institut Jacques Monod Paris France
Data accessibility	Repository name: Gene Expression Omnibus Data identification number: GSE164587 Direct URL to data: https://www-ncbi-nlm-nih-gov/geo/query/acc.cgi?acc=GSE164587
Related research article	T. Villa, M. Barucco, M.-J. Martin-Niclos, A. Jacquier, D. Libri, Degradation of non-coding RNAs promotes recycling of termination factors at sites of transcription, Cell Rep. 32 (2020) 107,942. https://doi.org/10.1016/j.celrep.2020.107942.

## Value of the Data

• Our data are important because they provide an assessment of the impact that expression of a circular ncRNA decoy exerts by out-titrating the NNS termination factor Nab3 and globally inhibiting its co-transcriptional binding to nascent target RNAs.
• The reported data will be beneficial to other researchers studying mechanisms of ncRNA transcription termination and may be useful in general for the design of strategies aimed at competitively inhibiting specific RNA-binding proteins.
• These data can be exploited to further investigate the relative affinity of nascent transcripts for Nab3 and NNS complex binding, and thus categorize different classes of RNAs on the basis of their responsiveness to NNS targeting. They can help conceive experiments to test the reactivity of diverse NNS-terminated RNA groups to different stresses and stimuli.

## 1 Data Description

**Fig. 1:**
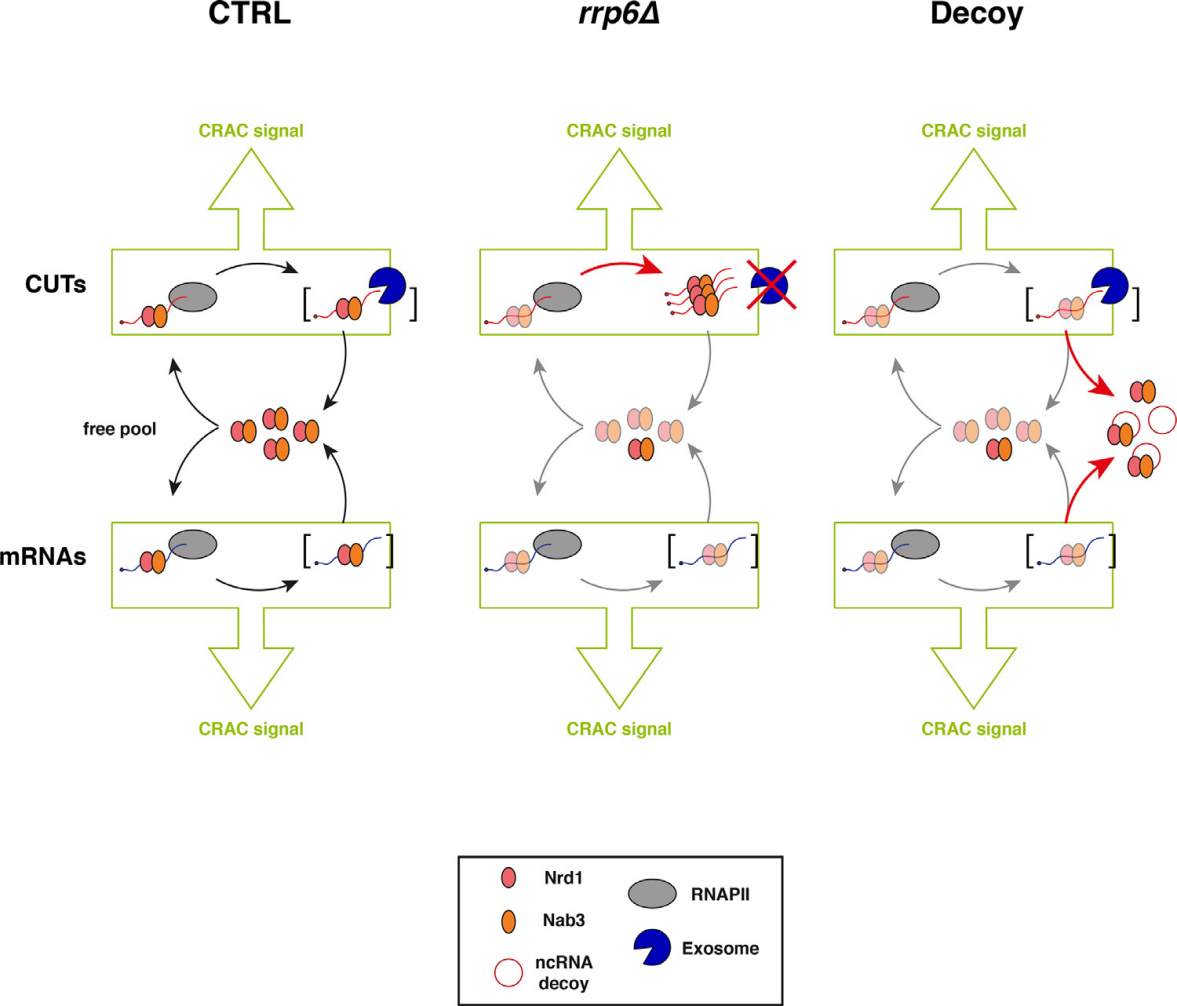
Schematic representation of the experimental rationale. As previously shown in the related research paper [1], out-titration of the Nrd1-Nab3 RNA-binding module by excess ncRNA accumulating in exosome-impaired *rrp6∆* cells or by expression of a circular ncRNA decoy leads to a decrease in its free pool (indicated by transparency). The overall CRAC signal is contributed by both co- and post-transcriptionally bound Nrd1-Nab3 to the RNA. Expression of the decoy is expected to decrease binding to nascent transcripts. Brackets indicate post-transcriptional complexes not expected to significantly contribute to the CRAC signal, possibly due to either fast export (mRNAs) or fast degradation (CUTs). Identity of schematized species is indicated in the boxed depiction.

**Fig. 2:**
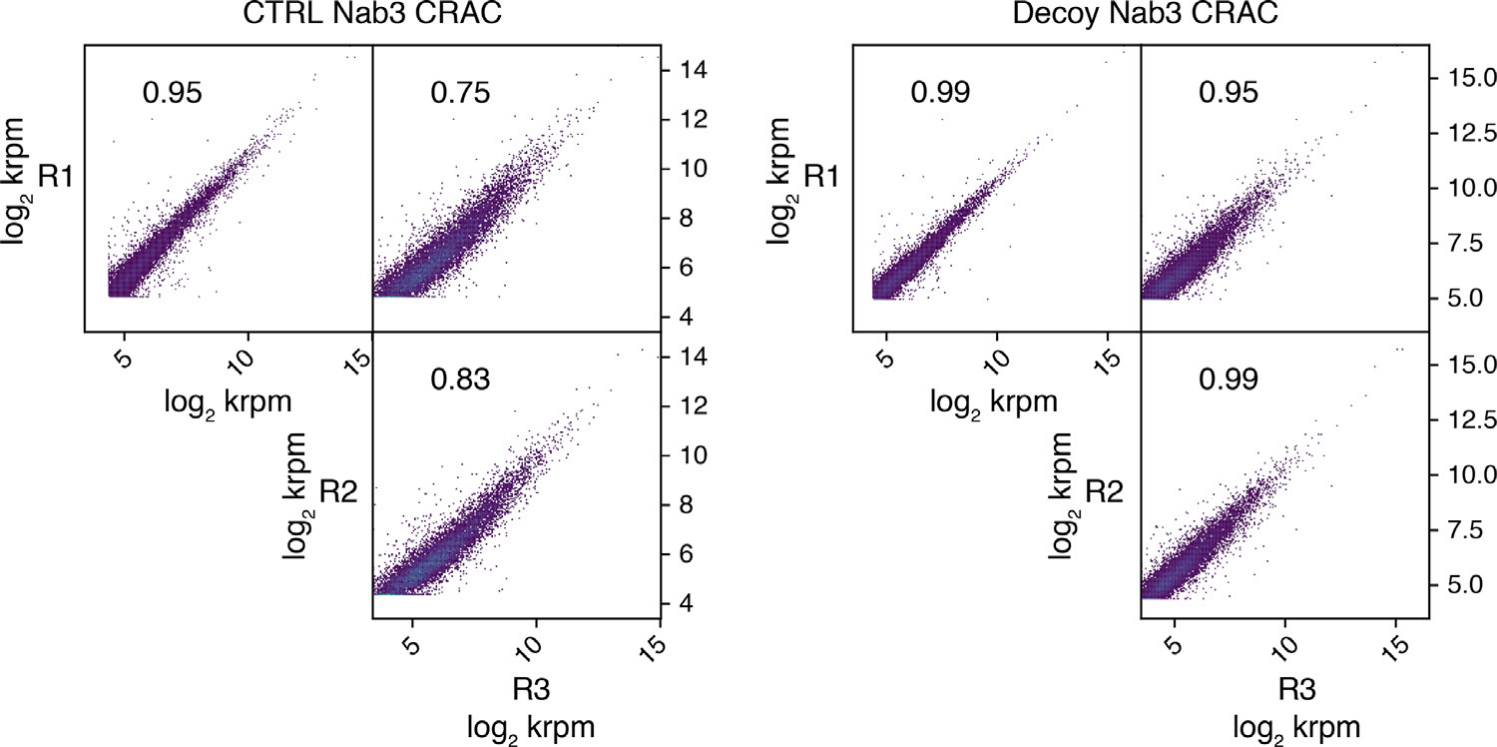
Correlation plots of the CRAC data. Dots represent 1 kb bins, plots were generated with the multiBigwigSummary and plotCorrelation Galaxy tools. For each comparison, the Pearson correlation coefficient is indicated.

**Fig. 3:**
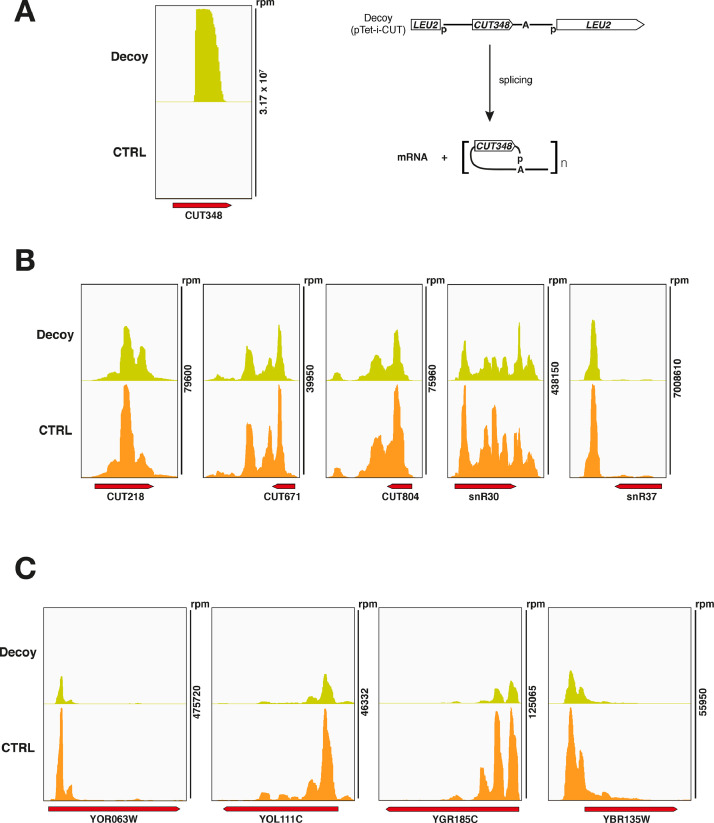
Read coverage determined by CRAC illustrating the binding of Nab3 to representative RNAs in the presence or absence of ncRNA decoy expression. (**A**) Nab3 signal at CUT348, the decoy sequence inserted in the pTet-i-CUT construct, as depicted on the right. Signal derives mostly from circular ncRNA decoy expressed from plasmid. (**B**) Binding of Nab3 to representative typical NNS targets such as Cryptic Unstable Transcripts (CUTs) and snoRNAs. (**C**) Binding of Nab3 to representative mRNAs. In all panels total hit densities per million mapped reads are indicated (scale shown on the right).

**Fig. 4:**
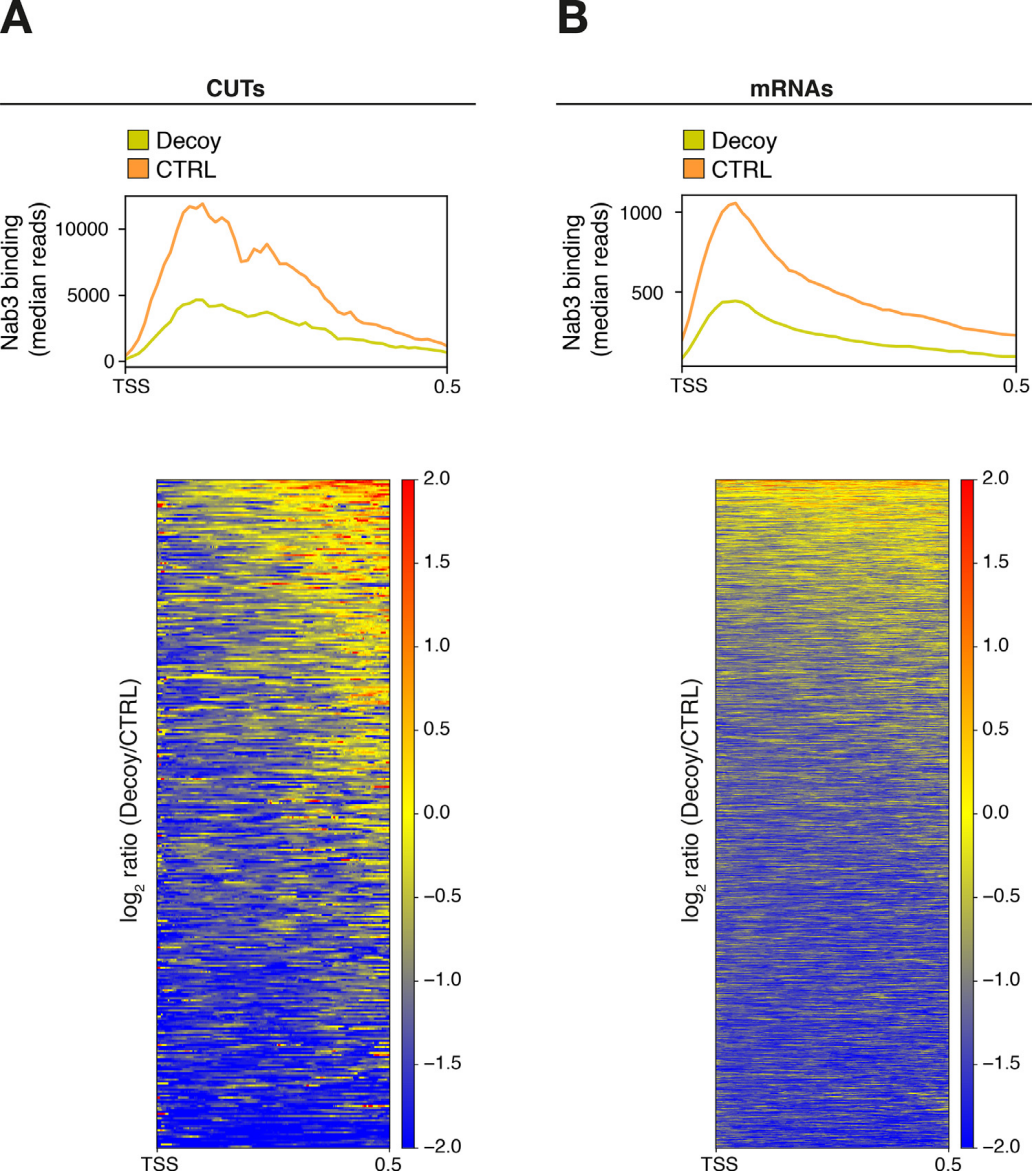
Metagene analysis of Nab3 binding. Metagene analyses showing the median Nab3 binding (top) and heatmaps illustrating the fold change (log_2_ Decoy/CTRL) distribution of Nab3 signal (bottom) on CUTs (**A**) or mRNAs (**B**). Features were aligned on the TSS, and in the heatmaps sorted by decreasing Nab3 average signal within the first 500 nucleotides downstream of the TSS.

We have recently shown that the transcription termination control that the Nrd1-Nab3-Sen1 (NNS) complex exerts on pervasive nuclear non-coding RNAs (ncRNAs), ultimately leading to their degradation by the nuclear RNA exosome, can be impaired by the sequestration of termination factors by accumulation of ncRNAs [1]. We have shown that this build-up of ncRNAs out-titrating the Nrd1 and Nab3 NNS RNA-binding proteins and preventing their efficient recycling back to elongating RNA Polymerase II (RNAPII) could be induced either by inhibition of the exosome in yeast *rrp6Δ* cells or by expression of a circular ncRNA decoy (Fig. 1). While in the related research paper we were able to show genomewide NNS-dependent termination impairment upon decoy expression through RNA-seq analysis, we were still missing direct proof that titration is occurring. Here, we present data from the analysis of nucleotide-resolution genomewide mapping of HTP-tagged Nab3 RNA binding in yeast cells expressing the circular ncRNA decoy designed to sequester Nab3 or a control construct [1]. Cells expressing the pTet-i-CUT and pTet-i constructs were pre-grown in the presence of doxycycline (i.e. under non-activating conditions), then expression of the constructs was induced upon release of doxycycline inhibition and allowed to reach mid-log phase when cells were subjected to *in vivo* UV crosslinking, and finally collected to be processed by the crosslinking and cDNA analysis (CRAC) protocol [2], [3], [4]. To allow normalization of the three independent biological replicates analyzed, cell lysates at the first step of the CRAC procedure were spiked-in with 8% of *S.pombe* lysates containing HTP-tagged RNAPII. After single-end sequencing of cDNA libraries, subsequent raw reads filtering, and mapping to reference yeast genome (R64), the normalized datasets (available at Gene Expression Omnibus NCBI repository, under data identification number GSE164587) were analyzed using deeptools 2.0 [5] on the Freiburg Galaxy platform (https://usegalaxy.eu/). Analysis of the correlation among biological replicates revealed significant Pearson correlation coefficients (Fig. 2). Next, read coverage determined by CRAC illustrating binding of Nab3 in the presence or absence of ncRNA decoy expression was analyzed. We first monitored binding to CUT348, the decoy sequence present in construct pTet-i-CUT, which revealed the expected very high binding (Fig. 3A). Then, we looked at representative typical NNS ncRNA targets such as Cryptic Unstable Transcripts (CUTs) and snoRNAs (Fig. 3B), as well as representative mRNAs (Fig. 3C). In all cases, binding of Nab3 appeared reduced in cells expressing the decoy. Finally, to extend the analysis of Nab3 binding to a genomewide perspective we generated metaprofiles and corresponding heatmaps illustrating the differential distribution of Nab3 CRAC signal between decoy expressing and control cells over CUTs (Fig. 4A) and mRNAs (Fig. 4B) aligned to their Transcription Start Site (TSS). In both cases, a clear global decrease in Nab3 binding was observed at a very large number of features.

## 2 Experimental Design, Materials and Methods

### 2.1 Yeast cultures

Yeast *S.cerevisiae* strain DLY3402 (*MATa NAB3::HTP::TRP1 dbr1∆::HIS3 ura3–1 ade2–1 leu2–3112 can1–100*) carrying either the control pTet-i or the decoy pTet-i-CUT constructs (plasmids pDL865 and pDL866, respectively; [1]) under control of the Tet_OFF_ promoter were grown in CSM-URA-TRP medium supplemented with 2 μg/ml Doxycycline at 30 °C to mid-log phase, back diluted to OD_600_ of 0.05 in 2 L CSM-URA-TRP medium without Doxycycline and grown to OD_600_ of 0.6 at 30 °C before being subjected to UV crosslinking *in vivo* and subsequently processed for CRAC. Yeast *S.pombe* strain DLP01 (*h90 Rpb1::HTP::kan^R^MX ura4-DS/E ade6-M210 leu1–32 mat3M::ura4+*) was grown in EMM+5AA medium to OD_600_ of 0.5 at 30 °C before being subjected to UV crosslinking *in vivo* and subsequently processed for CRAC.

### 2.2 Crosslinking and analysis of cDNAs (CRAC)

CRAC was performed exactly as described in the related research paper [1], with one notable modification: at the first step of the procedure cell lysates were spiked-in with 8% of *S.pombe* lysates containing HTP-tagged RNAPII to allow better normalization of the three independent biological replicates analyzed.

### 2.3 CRAC dataset processing

CRAC datasets were analyzed as described previously [3]. CRAC samples were demultiplexed using the pyBarcodeFilter script from the pyCRACutility suite [6]. Subsequently, the 5′ adaptor (read at the 3′ end of reads) was clipped with Cutadapt (with parameters {m 10}; [7]) and the resulting insert quality-trimmed from the 3′ end using Trimmomatic rolling mean clipping [8] (window size = 5, minimum quality = 25). At this stage, PCR duplicates were collapsed using the pyCRAC script pyFastqDuplicateRemover by recognizing a 6 nucleotide random tag that is part of the 3′ linker (the 3′ linker is read first in the sequencing reaction). During demultiplexing, pyBarcodeFilter retains this information in the header of each sequence. This information is used at this stage to better discern between identical inserts and PCR duplicates of the same insert. The sequences were subsequently reverse complemented with Fastx reverse complement (part of the fastx toolkit, http://hannonlab.cshl.edu/fastx_toolkit/) and mapped to the R64 genome [9] with bowtie2 (-N 1) [10]. For the *S. pombe* spike in present in the sample, reads that did not map on *S. cerevisiae* were mapped on the *S. pombe* genome after filtering for reads shorter than 40 nucleotides to avoid cross mapping of *S. cerevisiae* reads on *S. pombe*. The amount of mapped spike-in reads was used for normalization purposes.

### 2.4 Metagene analysis

Normalized genomic data obtained from CRAC were analyzed on the Freiburg Galaxy platform (http://deeptools.ie-freiburg.mpg.de/) feeding bigwig files to deeptools 2.0 [5]. Features were aligned on the TSS and metagene plots based on the median Nab3 binding signal were generated. For heatmap analyses illustrating the fold change distribution of Nab3 signal (log_2_ Decoy/CTRL), features were sorted by decreasing Nab3 average signal within the first 500 nucleotides downstream of the TSS. For correlation plots, the multiBigwigSummary and plotCorrelation Galaxy tools were used and the Pearson correlation coefficient in 1 kb bins was calculated for each comparison.

## CRediT Author Statement

**Tommaso Villa:** Conceptualization, Methodology, Formal analysis, Investigation, Writing - Original draft preparation, Reviewing and Editing; **Yan Jaszczyszyn:** Methodology; **Domenico Libri:** Conceptualization, Methodology, Formal analysis, Investigation, Writing - Original draft preparation, Reviewing and Editing, Funding acquisition, Supervision.

## Declaration of Competing Interest

The authors declare that they have no known competing financial interests or personal relationships which have or could be perceived to have influenced the work reported in this article.
